# *Neisseria meningitidis* activates pyroptotic pathways in a mouse model of meningitis: role of a two-partner secretion system

**DOI:** 10.3389/fcimb.2024.1384072

**Published:** 2024-09-23

**Authors:** Chiara Pagliuca, Roberta Colicchio, Silvia Caterina Resta, Adelfia Talà, Elena Scaglione, Giuseppe Mantova, Leonardo Continisio, Caterina Pagliarulo, Cecilia Bucci, Pietro Alifano, Paola Salvatore

**Affiliations:** ^1^ Department of Molecular Medicine and Medical Biotecnologies, University of Naples "Federico II", Naples, Italy; ^2^ Department of Biological and Environmental Sciences and Technologies, University of Salento, Lecce, Italy; ^3^ Department of Public Health, Experimental and Forensic Medicine, University of Pavia, Pavia, Italy; ^4^ Department of Science and Technology, University of Sannio, Benevento, Italy; ^5^ Department of Experimental Medicine, University of Salento, Lecce, Italy; ^6^ The Institute CEINGE-Biotecnologie Avanzate Franco Salvatore s.c.ar.l., Naples, Italy; ^7^ Task Force on Microbiome Studies, University of Naples "Federico II", Naples, Italy

**Keywords:** host-pathogen interaction, pyroptosis, Apoptosis, two-partner secretion systems, *Neisseria meningitidis*

## Abstract

There is evidence that in infected cells *in vitro* the meningococcal HrpA/HrpB two-partner secretion system (TPS) mediates the exit of bacteria from the internalization vacuole and the docking of bacteria to the dynein motor resulting in the induction of pyroptosis. In this study we set out to study the role of the HrpA/HrpB TPS in establishing meningitis and activating pyroptotic pathways in an animal model of meningitis using a reference serogroup C meningococcal strain, 93/4286, and an isogenic *hrpB* knockout mutant, 93/4286Ω*hrpB*. Survival experiments confirmed the role of HrpA/HrpB TPS in the invasive meningococcal disease. In fact, the ability of the *hrpB* mutant to replicate in brain and spread systemically was impaired in mice infected with *hrpB* mutant. Furthermore, western blot analysis of brain samples during the infection demonstrated that: i. *N. meningitidis* activated canonical and non-canonical inflammasome pyroptosis pathways in the mouse brain; ii. the activation of caspase-11, caspase-1, and gasdermin-D was markedly reduced in the *hrpB* mutant; iii. the increase in the amount of IL-1β and IL-18, which are an important end point of pyroptosis, occurs in the brains of mice infected with the wild-type strain 93/4286 and is strongly reduced in those infected with 93/4286Ω*hrpB*. In particular, the activation of caspase 11, which is triggered by cytosolic lipopolysaccharide, indicates that during meningococcal infection pyroptosis is induced by intracellular infection after the exit of the bacteria from the internalizing vacuole, a process that is hindered in the *hrpB* mutant. Overall, these results confirm, in an animal model, that the HrpA/HrpB TPS plays a role in the induction of pyroptosis and suggest a pivotal involvement of pyroptosis in invasive meningococcal disease, paving the way for the use of pyroptosis inhibitors in the adjuvant therapy of the disease.

## 1 Introduction

Two-partner secretion (TPS) systems are secretion pathways used by many pathogenic Gram-negative bacteria for the secretion of large proteins involved in the interaction between these bacteria and their hosts, and in inter-bacterial competition or cooperation. These systems rely on an exoprotein harboring an N-terminal secretion domain (generally termed TpsA) and a channel-forming β-barrel activator/transporter protein (termed TpsB) required for transport of the exoprotein across the outer membrane (Henderson et al., 2004; Jacob-Dubuisson et al., 2013; Schielke et al., 2010; Willett et al., 2015). The genome of *Neisseria meningitidis* strains may encode up to three different TPS systems (system 1 to system 3), and each of these systems may secrete multiple TpsA proteins (van Ulsen et al., 2008). The system 1 is present in all meningococci, while the systems 2 and 3 are over-represented in invasive isolates compared to carriage isolates but are present also in *Neisseria lactamica* (van Ulsen et al., 2008).

The meningococcal TPS system 1 (TpsA1/TpsB1, also known as HrpA/HrpB) has been involved in diverse functions. It is functions as a toxin/antitoxin fratricide system involved in intra-species competition (Arenas et al., 2013), and also plays a role in the interaction between meningococci and host cells through multiple mechanisms. In particular, it has been shown that HrpA is proteolytically processed to a ca. 180 KDa form and secreted via HrpB, and that a small amount of HrpA remains associated with the bacterial surface and contributes to the interaction with epithelial cells (Schmitt et al., 2007). Moreover, the secreted HrpA has also been implicated in biofilm formation on human bronchial epithelial cells (Neil and Apicella, 2009). Finally, there is evidence that HrpA plays a key role in intracellular survival by helping bacteria escape from the internalization vacuole into the cytoplasm, thus avoiding lysosomal killing (Talà et al., 2008b). Consistent with these findings, *hrpB* and *hrpA* mRNA levels increase in the intracellular environment, upon contact with host cells, and in anaerobiosis (Neil and Apicella, 2009; Talà et al., 2008b).

HrpA is a multi-domain, multi-tasking protein. A recent study demonstrates that while the carboxy-terminal region of HrpA acts as cell lysin that allows the bacteria to escape the internalization vacuole, the HrpA middle domain can bind the dynein light-chain, Tctextype 1 (DYNLT1) (Talà et al., 2022). The escape of meningococci into the cytosol and their interaction with the dynein motor led to inhibition of apoptosis and stimulation of pyroptosis (Talà et al., 2022), a form of pro-inflammatory cell death that could be implicated in the massive inflammation characteristic of invasive meningococcal disease (Man et al., 2017; Jiang et al., 2020). Indeed, in *in vitro* infected cells, *N. meningitidis* appears to suppress apoptosis by translocating the porin PorB into the mitochondrial membrane (Massari et al., 2000, 2003, 2010; Linhartova et al., 2006; Tunbridge et al., 2006) and induce pyroptosis by non-canonical pathway that requires the exposure or release of the lipooligosaccharide (LOS) into the cytosol (Talà et al., 2022; Idosa et al., 2019).

Since all information on the possible physiological role of HrpA/HrpB TPS was obtained through *in vitro* studies with immortalized cell lines (Talà et al., 2008b, 2022), here we set out to study the role of HrpA/HrpB TPS in establishing invasive meningococcal disease in a suitable animal model. We used a model of meningococcal meningitis based on intracisternal infection of adult mice (Colicchio et al., 2009), which was previously validated by using the reference serogroup C meningococcal strain 93/4286 and its isogenic unencapsulated mutant (Colicchio et al., 2019). This mouse model induces both primary meningitis and invasive meningococcal disease compared to intraperitoneal and intranasal models, which are generally less suitable to study meningitis and central nervous system (CNS) infection, as animals may die of sepsis before the onset of meningitis. In this animal model, meningococcal replication in the brain is very efficient, although meningococci are also found in the blood and in peripheral organs in particular spleen and liver of infected mice (Colicchio et al., 2009, 2019). At sublethal doses meningococci multiply in brain, spleen and liver, while they are unable to multiply in the blood (Colicchio et al., 2009). Bacteria persist in the liver and this behavior was imputed to response of bacteria to iron-based nutritional immunity (Letendre and Holbein, 1984), since the liver with its large pool of ferritin represents a target peripheral organ for replication of meningococci that can obtain iron from ferritin (Letendre and Holbein, 1984; Larson et al., 2004). In the brain, meningococci are massively found in the *corpus callosum*, and also associate with the cells lining the ventricles and *choroid plexus* (Colicchio et al., 2019). In mice infected via intracisternal injection, the *choroid plexus*, which is an important gateway for meningococcal traversal from the bloodstream into the cerebrospinal fluid (CSF) during meningitis in humans (Schwerk et al., 2015) and plays a crucial role in CSF production and iron homeostasis in the brain (Mesquita et al., 2012; Rouault et al., 2009), can be used by meningococci to multiply actively and to spread systematically using a reverse path (Colicchio et al., 2019).

Using the mouse model, we will try to answer the following questions: is the HrpA/HrpB TPS system a virulence determinant in the animal model of meningitis and systemic infection? Is the system necessary for efficient meningococcal replication in the host tissues and for systemic spread of bacteria? Is the HrpA/HrpB TPS system essential for activation of pyroptotic pathways during invasive meningococcal disease?

## 2 Materials and methods

### 2.1 Bacterial strains and growth conditions

In this study serogroup C meningococcal strains have been used: 93/4286 reference strain belonging to the ET-37 hypervirulent lineage (CC ST-11), and 93/4286Ω*hrpB* isogenic mutant *hrpB*-deficient. Bacteria were cultured on gonococcus (GC) agar or broth (Oxoid S.p.A., Milan, Italy) supplemented with 1% (vol/vol) Polyvitox (Oxoid) at 37°C with 5% CO_2_. Erythromycin (Sigma-Aldrich/Merck KGaA, Darmstadt, Germany) was added to a final concentration of 7 µg ml^-1^, when needed.

Growth/survival rates were evaluated for each strain, at 0.5 O.D., 1.0 O.D. and until the third hour post reaching 1.0 O.D., by plating serial dilutions on GC agar in presence or absence of erythromycin, incubated at 37°C with 5% CO_2_ for 24 h and viable counts were determined by CFU methods.

The growth rate µ (h^-1^) of the *hrpB*-defective mutant and the wild-type strain was calculated as previously described (Hall et al., 2014). All experiments were performed in triplicate.

For mouse challenge the inocula were prepared by cultivating bacteria in GC broth until mid-logarithmic phase. Viable cell counts were determined, and bacteria were frozen at -80°C with 10% glycerol until use. The DH5α *Escherichia coli* strain was used in cloning procedures. The bacteria were grown in Luria-Bertani (LB) (Oxoid) medium. LB medium was supplemented with ampicillin (50 µg ml^-1^) (Sigma-Aldrich/Merck KGaA), to allow plasmid selection.

### 2.2 DNA procedures and *N. meningitidis* transformation

High-molecular-weight genomic DNA from *N. meningitidis* strains was extracted and recovered as previously reported (Bucci et al., 1999; Sambrook and Russell, 2001). Oligonucleotide synthesis and DNA sequencing were performed by CEINGE-Advanced Biotechnologies, Naples, Italy.

DNA sequence analysis was carried out by using the GeneJockey Sequence Processor software (Biosoft) and the multiple-sequence alignment tool ClustalW (http://www.ebi.ac.uk/Tools/msa/clustalw2/).

To construct the pDEΔhrpB plasmid, a 754 bp-long *hrpB* gene fragment was PCR amplified from genomic DNA of *N. meningitidis* 93/4286 using the primer pair hrpBfor (5’- GTAACTTCTGGATCCGCTTTCAGTTTCCGTACCGGTGGC-3’) and hrpBrev (5’- CAGCACATGGATCCTGAATTGTTAACTGATGCAAATGTC-3’). PCR conditions were as follows: 45 s of denaturation at 94°C, 45 s of annealing at 65°C, and 60 s of extension at 72°C for a total of 30 cycles. Reactions were carried out in a MyCycler thermal cycler (Bio-Rad, Laboratories S.r.l., Segrate, Milan, Italy). The amplicon was cloned into the BamHI site of *Neisseria*-*E. coli* shuttle plasmid pDEX (Colicchio et al., 2009, 2019).

pDEΔ*hrpB* plasmid was used to genetically inactivate *hrpB* gene (old locus tag NMC0443 and locus tag NMC_RS02405) in *N. meningitidis* 93/4286 by single crossover. Transformation experiments were performed using 0.5 to 1 µg of plasmid DNA as previously described (Bucci et al., 1999; Pagliarulo et al., 2004). Transformants were selected on GC agar medium supplemented with erythromycin (7 µg ml^-1^). Successful gene inactivation was proven by Southern blot hybridization using the 754 bp-long *hrpB*-specific ^32^P-labeled gene fragment as a probe. Southern blot hybridization was performed according to standard procedures (Sambrook and Russell, 2001). ^32^P-labeling of DNA fragments was carried out by random priming using the Klenow fragment of *E. coli* DNA polymerase I and (α-^32^P) dGTP (3,000 Ci mmol-1) (Sambrook and Russell, 2001).

### 2.3 *In vivo* experimental design

Eight-week-old female inbred BALB/c mice weighing 19 to 20 g were obtained from Charles River Italia (Lecco, Italy). The animals were fed with laboratory food pellets and tap water *ad libitum* and were housed under specific-pathogen-free conditions. All attempts were made to minimize murine suffering and reduce the number of animals according to the European Communities Council Directive of 24 November 1986 (86/609/EEC). The study was approved by the Ethical Animal Care and Use Committee (protocol number 2, 14 December 2012) and the Italian Ministry of Health (protocol number 0000094-A-03/01/2013).

Mice were infected through the intracisternal route, as previously described (Colicchio et al., 2009, 2019). Meningococci for mouse challenge were prepared as previously reported (Colicchio et al., 2009, 2019). Briefly, bacteria were thawed, centrifuged for 15 min at 1,500 x g, and suspended in GC broth with iron dextran (5 mg/kg; Sigma-Aldrich/Merck KGaA). Approximately 2 h before intracisternal infection, mice were injected intraperitoneally with iron dextran (250 mg kg^-1^). Animals were lightly anesthetized (50 mg/kg ketamine and 3 mg/kg xylazine or Zoletil 20 (30 mg/kg; VirbacSrl) and Xilor (8 mg/kg; Bio 98 Srl)), and bacteria (suspended in a total volume of 10 µl) were inoculated by hand puncturing the cisterna magna of mice using a 30-gauge needle (BD Italia, Milan, Italy). Mice were monitored for possible seizures due to inoculation practice. According to a previously described coma scale (Liechti et al., 2014), clinical signs were monitored and mice with a score of 2 were euthanized and recorded as dead for statistical analysis.

### 2.4 Animal survival and CFU counts

Inbred BALB/c mice (8 weeks old) were infected by the intracisternal route, as described above. Two groups of mice were infected with the wild-type strain *N. meningitidis* 93/4286 (6 mice/dose; 10^5^ or 10^6^ CFU per mouse) or with the isogenic mutant 93/4286Ω*hrpB* (7 mice/dose; 10^6^ or 10^7^ CFU per mouse), respectively. Control group was inoculated with GC broth alone. Mice were monitored for clinical signs, body weight and temperature throughout the experiment, as described previously (Colicchio et al., 2009). Survival was recorded for 60 h. To compare the virulence of 93/4286 wild-type strain versus that of the *hrpB*-defective mutant, two groups of mice (n. 3/group/time points) were infected with a sub-lethal dose and sacrificed at different time points (12, 24 and 48 h) after challenge for organ collection, as previously reported (Colicchio et al., 2019). Excised organs (brain, spleen, and liver) were homogenized in 1 ml of GC broth. Viable cell counts were performed by plating 10-fold dilutions on GC agar plates and on GC agar supplemented with erythromycin for the mutant strain.

### 2.5 Cell culture and gentamycin protection assay

THP-1 monocytes were grown in RPMI-1640 medium supplemented with 10% fetal bovine serum (FBS), 2 mM L-glutamine, 50 U/mL penicillin and 50 μg/mL streptomycin. Infection with 93/4286 or 93/4286Ω*hrpB* strains was conducted, as previously described (Talà et al., 2022), on THP-1 after treatment with 2 nM of PMA for 5 days to differentiate them into macrophages. Briefly, cells were infected with a multiplicity of infection (MOI) of 1:100, after 1 h gentamycin at 100 μg/ml was used to kill the extracellular bacteria and infection was stopped at different time points (3, 5 and 7 h). The survival/growth of extracellular bacteria was evaluated by plating serial dilutions of the cell media on GC agar and counting the CFU the day after. The plating of the cell lysate in 0,1% saponin on GC agar allows intracellular bacteria survival/growth evaluation.

### 2.6 Western blotting

After 12, 24 and 48 h of infection with *N. meningitidis* 93/4286 strain or 93/4286Ω*hrpB* isogenic mutant, mice were sacrificed, brains and spleens were collected and homogenized as described. THP-1 macrophages were infected with the same strains for 3, 5 and 7 h. Mouse homogenates and THP-1 cells were lysed with Laemmli Buffer (100 mM Tris–HCl, pH 6.8, containing 4% SDS, 20% glycerol and 0.2% blue bromophenol) for their subsequent use in sodium dodecyl sulphate - polyacrylamide gel electrophoresis (SDS-PAGE), as previously reported (Talà et al., 2022). Briefly, samples were loaded onto polyacrylamide gels and then transferred onto polyvinylidene difluoride (PVDF) or nitrocellulose membrane. These were subsequently blocked with 5% non-fat dry milk in phosphate buffered saline (PBS)-Tween 0.1% and incubated with antibody against caspase-1 (abcam, ab179515), caspase-11 (abcam, ab22684), caspase-7 (Cell Signaling Technology, #9492), gasdermin-D (Cell Signaling Technology, #39754), caspase-3 (Cell Signaling Technology, #9662), IL-1β (Invitrogen, #P420B), IL-18 (abcam, ab207323) or TNF-α (Invitrogen, #AMC3012). Secondary antibodies used were horseradish peroxidase (HRP)-conjugated anti-mouse monoclonal antibody and HRP-conjugated anti-rabbit monoclonal antibody (Invitrogen). Clarity and Clarity Max ECL Western Blotting Substrate (Bio-Rad) were used to visualize the blots that were analyzed by Image Lab™ software version 6.0.1. Data were reported as mean protein expression of mice tissue infected with mutant strain ± standard error respect to expression in non-infected mouse or wild-type infected mouse.

### 2.7 Statistical analysis

To evaluate differences in *in vitro* growth rates and in bacterial loads between mice infected with the wild-type or mutant strains, Student’s *t* test was performed. Animal survival was estimated by Kaplan-Meier survival analysis. Bacterial CFU from excised organs and time points were represented as geometric means ± standard deviations (SD) of log CFU from individual mice. GraphPad Prism (version 6) software was used to perform two-way ANOVA with Dunnett *post hoc* test to assess differences in the expression levels of the selected markers between 93/4286Ω*hrpB* and 93/4286 infected mice brains. THP-1 and mice infections were repeated at least three times, data were represented as the mean ± SEM and analyzed through *t*-test (*p<0.05, **p<0.01, ***p<0.001).

## 3 Results

### 3.1 Generation and characterization of *hrpB*-defective isogenic mutant in serogroup C

**Figure 1:**
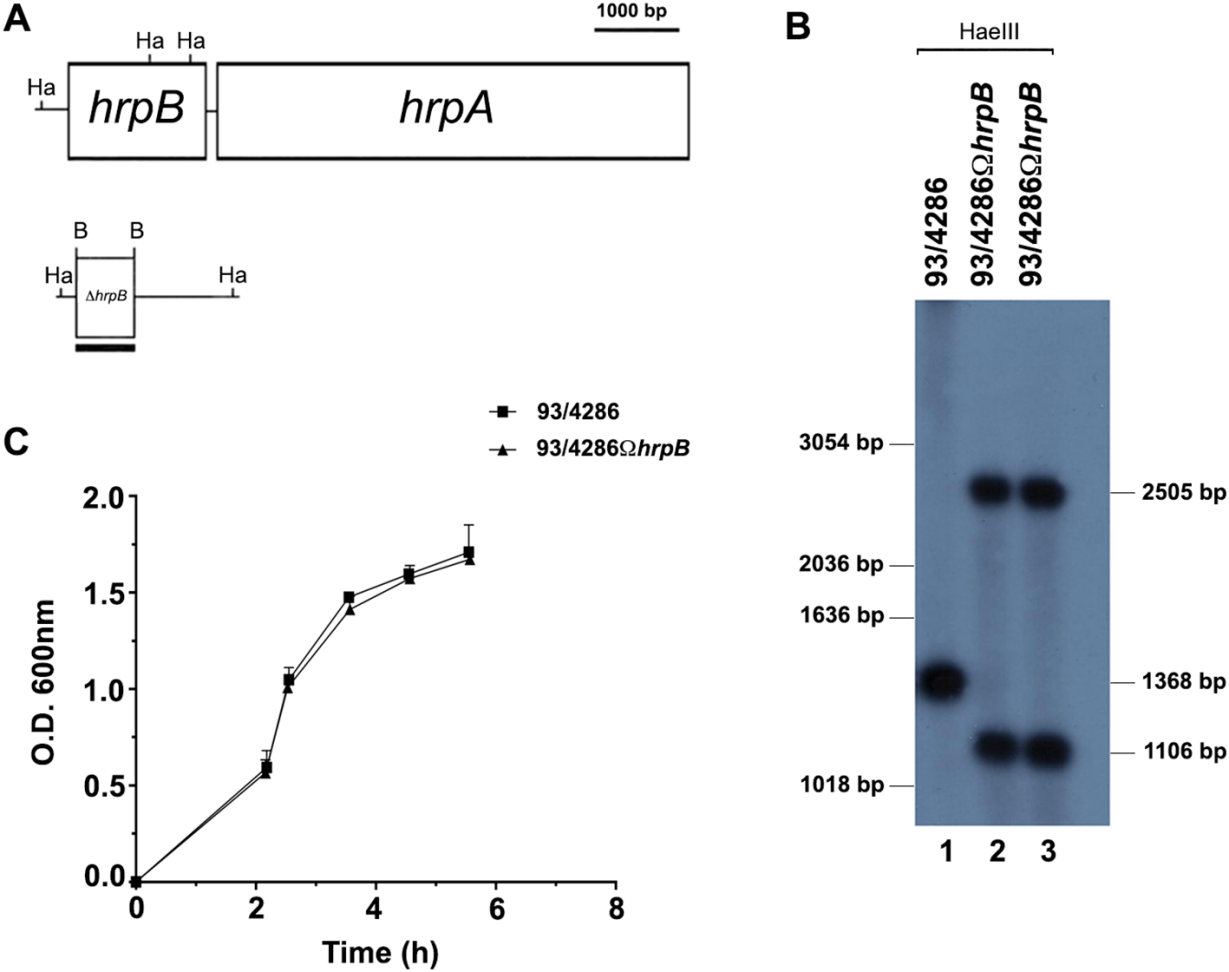
Construction and characterization of *hrpB*-defective mutant. **(A)** Experimental design for *hrpB* disruption by single crossover. The genetic map of the *hrpB* locus of *N. meningitidis* serogroup C was constructed on the basis of the available nucleotide sequences of FAM18 (ET-37) in the NCBI data bank (accession number NC_008767). **(B)** Southern blot analysis demonstrating the inactivation of the *hrpB* gene. Chromosomal DNA was extracted from the parental strain 93/4286 (lane 1) and the isogenic mutants 93/4286Ω*hrpB* (lane 2, 3) obtained by transformation of 93/4286 with pDEΔ*hrpB* and selection with erythromycin. Chromosomal DNA was analyzed by Southern blot hybridization using a *hrpB*-specific probe. Bars on the right indicate *hrpB*-specific fragments whose sizes were deduced on the basis of the relative migration pattern of DNA ladders (bars on the left). **(C)** Growth curves of the wild-type and the *hrpB*-defective isogenic mutant in GC broth. The experiments were performed in triplicate with three independent cultures, and statistical significance was examined by the Student’s *t* test. Results are indicated as means ± SDs.

To investigate the pathogenetic role of HrpB/HrpA two partner secretion system in *in vivo* infection model, the 93/4286Ω*hrpB* mutant of the *N. meningitidis* serogroup C reference strain 93/4286 was obtained by insertional inactivation of the *hrpB* gene (locus tag NMC0443 in the reference strain FAM18 (serogroup C, ET-37), coding for the channel-forming β-barrel activator/transporter protein of the HrpA/HrpB TPS. In the meningococcal genome, *hrpB* is located upstream of *hrpA*, coding for secreted HrpA (**Figure 1A**).

Southern blot hybridization demonstrated the successful gene inactivation by using a *hrpB*-probe. Two HaeIII DNA fragments of the expected dimensions (2505 bp and 1106 bp) were revealed in the 93/4286Ω*hrpB* mutant strain, whereas only a single 1368 bp HaeIII fragment was detected in the 93/4286 wild-type strain (**Figure 1B**).

To exclude some differences during bacterial replication, the growth rates of the 93/4286 wild-type strain and the isogenic *hrpB* mutant were evaluated during the growth in GC broth at 37°C. The *hrpB* mutant showed growth curve similar to that of the wild-type strain, with a growth rate value (µ = 0.876 ± 0.230) equal to that of the wild-type strain (µ = 0.954 ± 0.302) without any statistically significant difference (**Figure 1C**).

### 3.2 Increased survival of mice infected with the 93/4286Ω*hrpB* mutant

**Figure 2:**
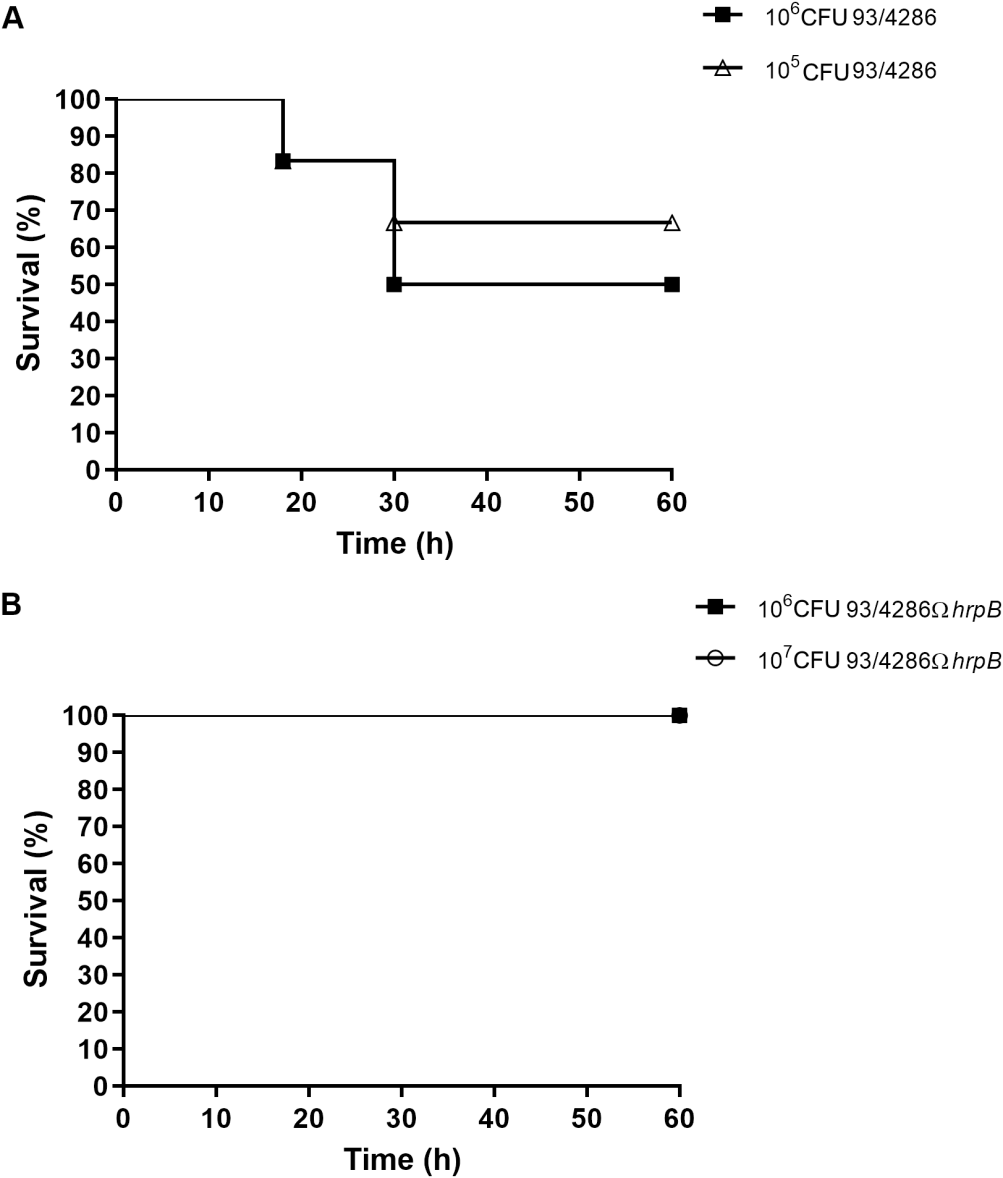
Survival in mice infected with either 93/4286 or 93/4286ΩhrpB meningococcal strains. **(A)** Two groups of BALB/c mice were infected by intracisternal injection of 10^5^ and 10^6^ CFU/mouse of *N. meningitidis* 93/4286 strain or **(B)** 10^6^ and 10^7^ CFU/mouse of the isogenic 93/4286Ω*hrpB* mutant. Mice were monitored for 60 h and survival was recorded. Results are expressed as percent of survival over time.

The virulence of the *hrpB*-defective strain was evaluated in the mouse model of experimental meningococcal meningitis (Morawietz et al., 2004; Ricci et al., 2014; Colicchio et al., 2019; Pagliuca et al., 2019) by analyzing the survival of the animals at different time points. To determine the lethal dose for 50% of animals (LD_50_), two groups of BALB/c mice were infected by intracisternal injection of either the wild-type strain *N. meningitidis* 93/4286 (6 mice/dose; 10^5^ or 10^6^ CFU/mouse) (**Figure 2A**) or the mutant strain 93/4286Ω*hrpB* (7 mice/dose; 10^6^ or 10^7^ CFU/mouse) (**Figure 2B**). As previously reported (Colicchio et al., 2019; Pagliuca et al., 2019), mouse death and weight loss occurred within the first 48 h after meningococcal injection. A significant difference was detected among the two groups of animals with 65% or 50% survival in mice inoculated, respectively, with 10^5^ CFU or 10^6^ CFU of the wild-type 93/4286 strain (**Figure 2A**), compared to 100% survival of rodents challenged with 10^6^ CFU or 10^7^ CFU of the mutant 93/4286Ω*hrpB* strain (**Figure 2B**). These data suggest that *hrpB* is required for virulence in the mouse model of meningococcal meningitis. Consistent with the results of the animal survival tests, weight loss was much more pronounced at 24 and 48 h after intracisternal inoculation in mice infected with 93/4286 (10^6^ CFU/mouse) compared with those infected with 93/4286Ω*hrpB* (10^7^ CFU/mouse) (**Supplementary Figure S1**).

### 3.3 Replication of the 93/4286Ω*hrpB* mutant is reduced in the murine brain

**Figure 3:**
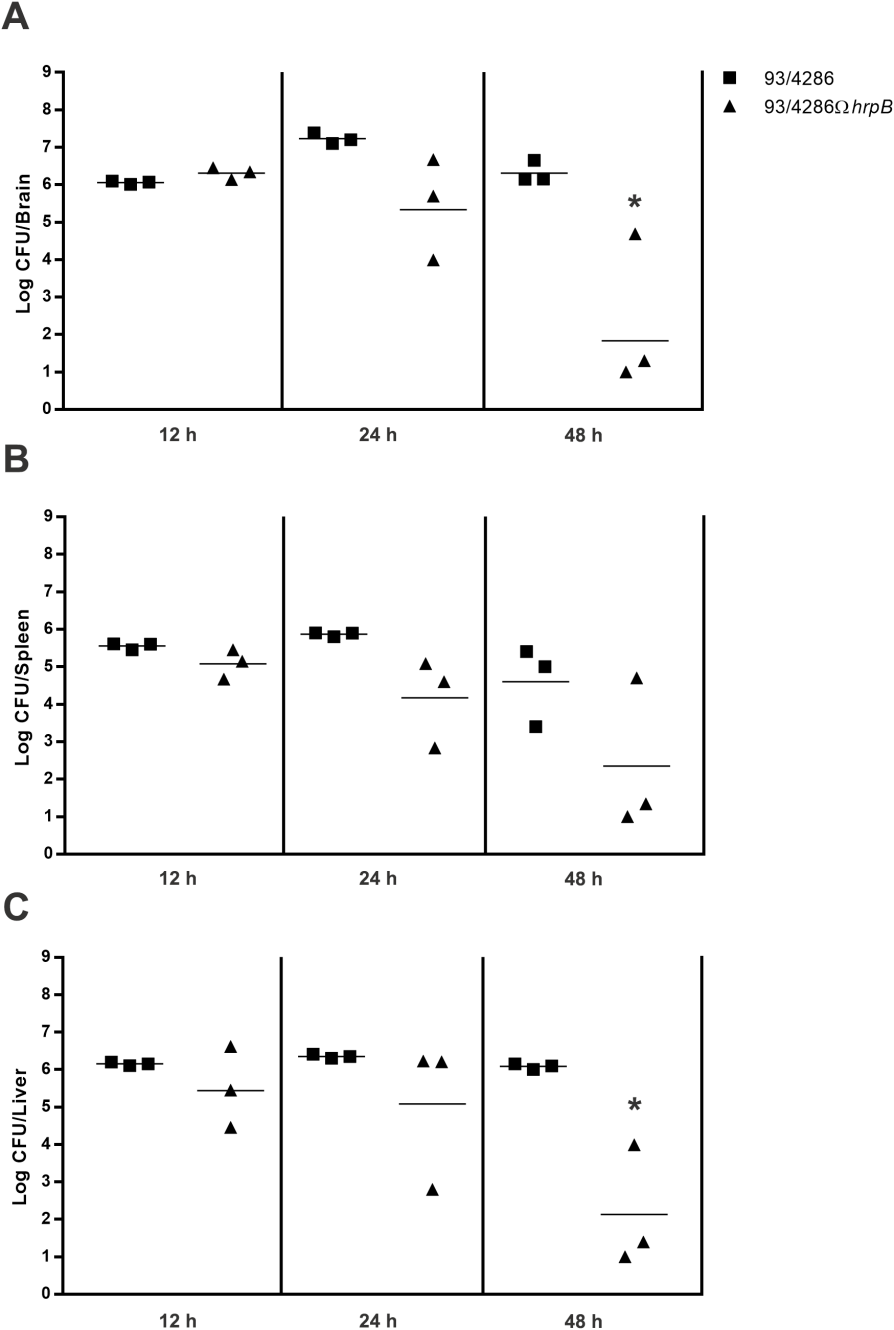
Bacterial loads in brains, spleen and liver of mice infected with either 93/4286 or 93/4286Ω*hrpB* meningococcal strains. Two group of BALB/c mice were infected by the intracisternal route (n=9/group) with sub-lethal dose of 93/4286 or 93/4286Ω*hrpB*. Animals were sacrificed at 12, 24 and 48 h after inoculation. Bacterial loads in brains **(A)**, spleen **(B)** and liver **(C)** of mice infected were recorded over time. The organs were collected and homogenized in GC medium and CFU were determined. Results are expressed as log CFU per organ. Horizontal bars indicate the geometrical means of CFU values. Each symbol represents a single animal.

To assess bacterial replication in the brain, mice were injected intracisternally with sub-lethal dose (5x10^5^ CFU) of the 93/4286 wild-types or the 93/4286Ω*hrpB* mutant strains and sacrificed at different time points after challenge to determine the CFU (12, 24 and 48 h) (**Figure 3A**). Three mice/group/time points were infected with 93/4286 strain or with 93/4286Ω*hrpB* mutant strain. Increased CFU counts was recorded for the wild-type bacteria that achieved the highest numbers 24 h after the intracisternal injection (7.320 ± 0.147 log CFU), consistent with previous findings (Colicchio et al., 2019). At 48 h the bacteria titers decreased slightly (6.399 ± 0.354 log CFU). In contrast, bacterial loads in the brain of mice challenged with the *hrpB*-defective mutant did not increase 24 h after the inoculation, and declined over time reaching 2.330 ± 2.049 log CFU at 48 h. Furthermore, bacterial clearance in the brain occurred in 50% of mice infected with the *hrpB*-defective mutant, and never occurred in mice infected with the wild-type strain (**Figure 3A**).

### 3.4 Clearance of the 93/4286Ω*hrpB* mutant is enhanced in the mouse liver and spleen

To evaluate the clearance of bacteria, the same animals sacrificed to assess replication in the mouse brain (9 mice infected with 93/4286, and 9 mice infected with 93/4286 Ω*hrpB*) were used to determine meningococcal viable counts in the spleen (**Figure 3B**) and liver (**Figure 3C**). Similar to the results obtained with mouse brain, bacterial clearance in the liver and spleen occurred in 50% of mice infected with the *hrpB*-defective mutant, and never occurred in mice infected with the wild-type strain. In the spleen of animals infected with wild-type strain, bacterial loads were similar at 12 h and 24 h (5.552 ± 0.088 and 5.866 ± 0.057 log CFU, respectively), and decreased slightly at 48 h (4.599 ± 1.060 log CFU) (**Figure 3B**). Bacterial titers in the liver of these animals remained high and almost unchanged 12, 24 and 48 h after the inoculation, respectively, 6.151 ± 0.048, 6.354 ± 0.056, and 6.083 ± 0.076 log CFU (**Figure 3C**). On the contrary, bacterial counts in the liver and spleen of mice challenged with the *hrpB*-defective mutant progressively declined over time reaching 2.238 ± 1.834 log CFU at 48 h.

### 3.5 *N. meningitidis* activates pyroptotic pathways in the mouse brain, and their activation is reduced in mice infected with the 93/4286Ω*hrpB* mutant

**Figure 4:**
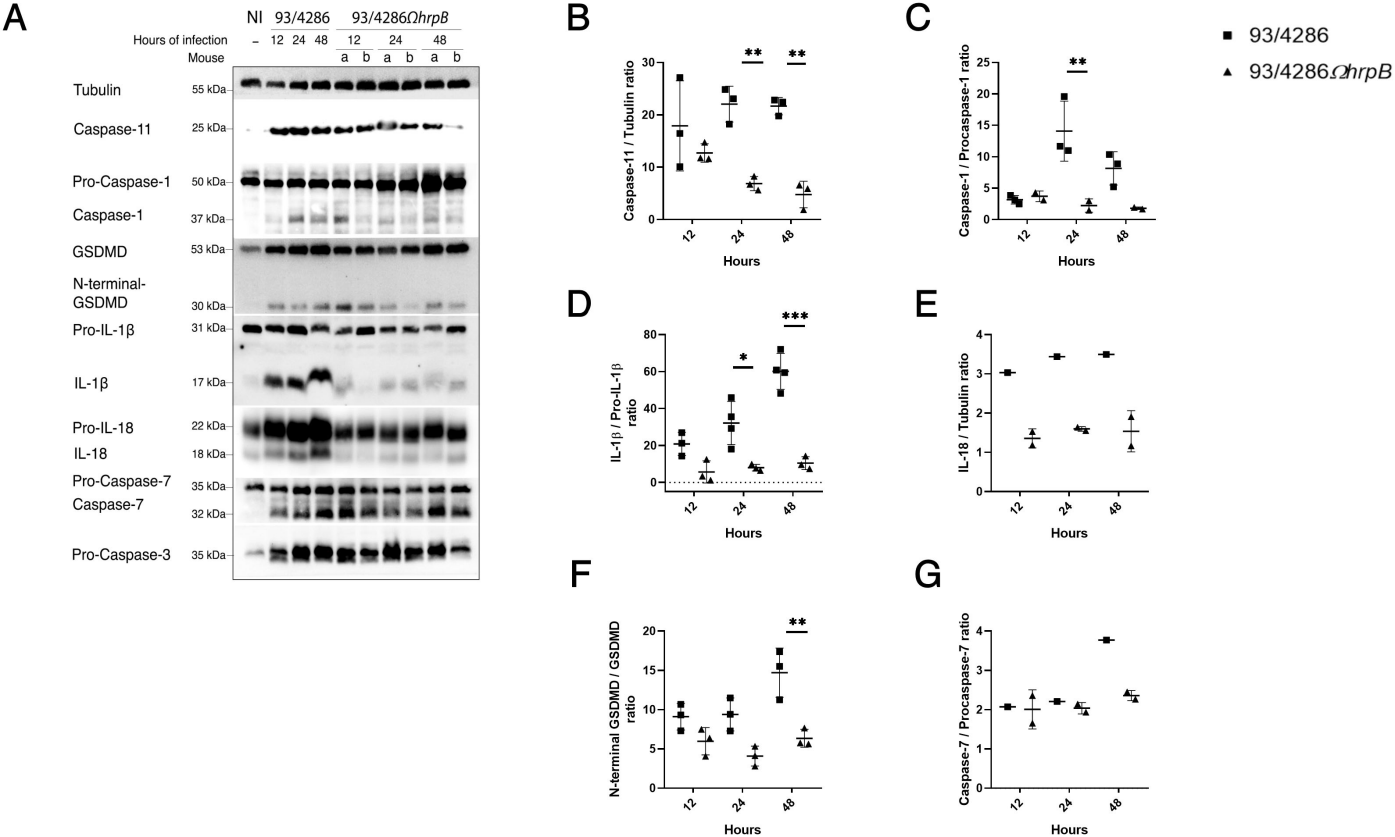
Pyroptosis markers expression in mouse brains after infection with either 93/4286 or 93/4286Ω*hrpB* meningococcal strains. **(A)** Mice were infected with 93/4286 or 93/4286Ω*hrpB* strain and sacrificed after 12, 24 and 48 h of infection. Three mice for each time point were infected with 93/4286, and three mice for each time point were infected with 93/4286Ω*hrpB*. Brains were collected, homogenized, lysed, and analyzed by immunoblotting using antibodies against caspase-11, caspase-1, gasdermin-D (GSDMD), caspase-7, caspase-3, interleukin 1β (IL-1β) and IL-18. Anti-tubulin antibody was used as a loading control. **(B–G)** Densitometric analysis quantification is reported as fold change of values of infected brains compared to values of non-infected brains. The expression is reported as a scatter dot plot ± SD. Two-way ANOVA with Dunnett test was performed (*p<0.05, **p<0.01, ***p<0.001).

Mice were infected with 93/4286 or 93/4286Ω*hrpB* strain and sacrificed after 12, 24 and 48 h of infection. Brains were collected and lysed as described in Material and Methods section. Western blots analysis showed cleaved forms of caspase-1, caspase-11, gasdermin-D, caspase-7 and IL-1β only in infected mice, with differences in the expression between 93/4286-infected versus 93/4286Ω*hrpB*-infected mice (**Figure 4**; **Supplementary Figure S2**). In particular, in 93/4286 infected mice we observed increased activation of caspase-1, caspase-11, gasdermin-D, caspase-7, IL-1β and IL-18 during the course of infection with peak activation of caspase-1 and caspase-11 at 24 h and a slight decrease at 48 h (**Figures 4A–C**), while peak activation of gasdermin-D and caspase-7 was reached at 48 h (**Figures 4A, F, G**). Conversely, in 93/4286Ω*hrpB*-infected mice we observed activation of caspase-1, caspase-11, gasdermin-D and caspase-7 to the same extent as observed in 93/4286 infected mice at 12 h. After this time, however, progressive decrease in the amount of activated caspase-1 and caspase-11 at 24 h and 48 h was observed in these mice (**Figures 4A–C**), while the amount of activated gasdermin-D and caspase-7 did not change substantially (**Figures 4A, F, G**).

Comparatively, at the corresponding time points, we observed a reduction of caspase-1 cleavage in brains infected with 93/4286Ω*hrpB* after 24 and 48 h (by 84% ± 0.053 and 77,8% ± 0.017, respectively) compared to brains infected with 93/4286, while no changes were found after 12 h of infection (**Figures 4A, C**). The same trend was observed for the expression of cleaved caspase-11 (reduction of 68,8% ± 0.035 after 24 h and 77,8% ± 0.067 after 48 h in 93/4286Ω*hrpB*-infected mice compared to 93/4286 infected mice) (**Figures 4A, B**). The amounts of the cytotoxic N-terminal fragment of gasdermin-D showed a reduction of 56,4% ± 0.09 at 24 h and 56,83% ± 0.054 at 48 h in 93/4286Ω*hrpB*-infected mice compared to 93/4286 infected mice (**Figures 4A, F**). The amount of the caspase-7 subunit showed a reduction of 37% ± 0.024 at 48 h in mice infected with 93/4286Ω*hrpB* compared to mice infected with 93/4286, while no differences were observed at 12 h and 24 h in mice infected with one or the other strain (**Figures 4A, G**). It may be also noted that the active form of caspase-3 was not detected at any time during the infection, indicating that it is not processed and activated in this infection model (**Figure 4**).

**Figure 5:**
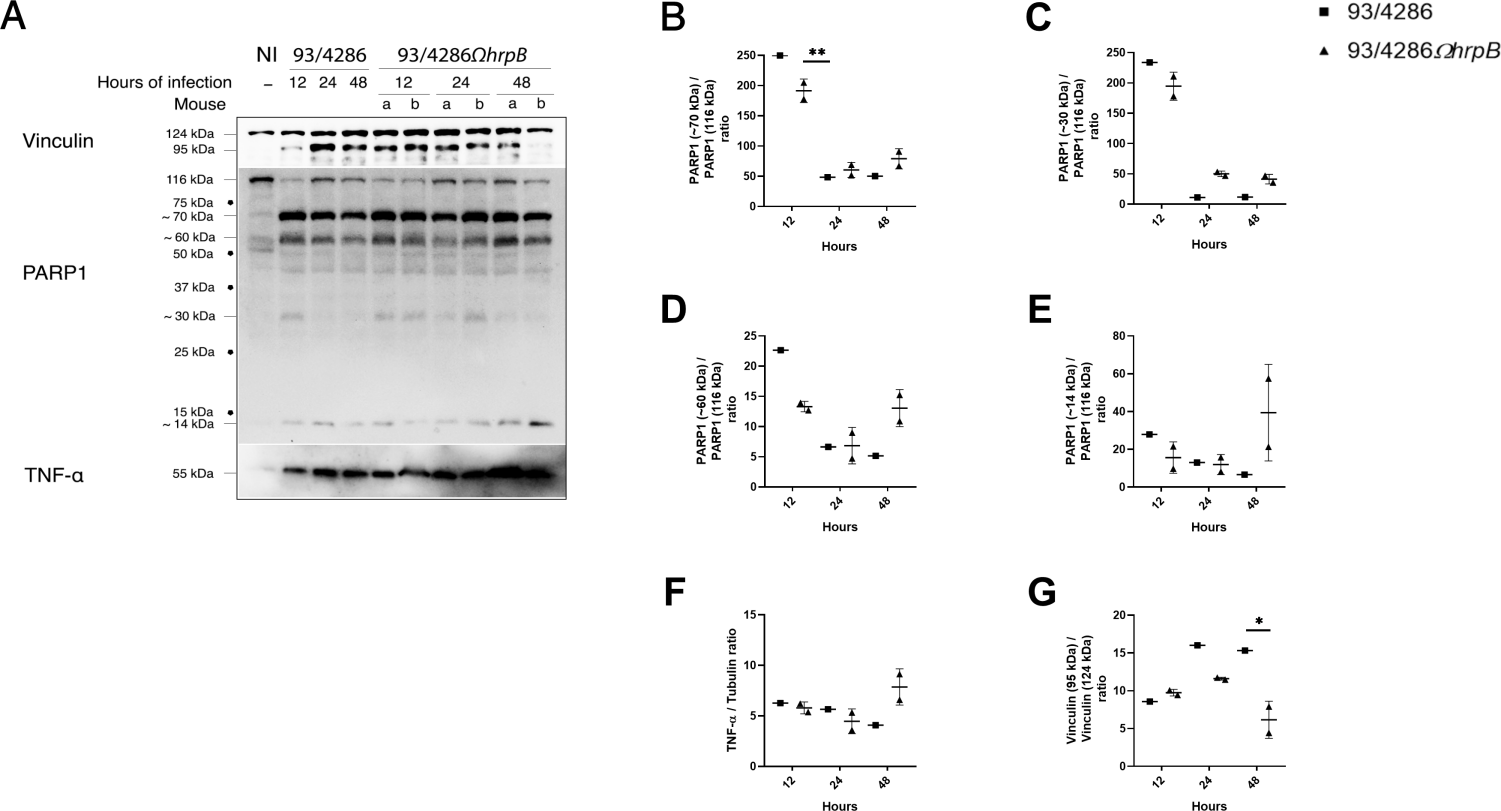
Vinculin, PARP-1 and TNF-α expression and/or fragmentation in mouse brains after infection with 93/4286 or 93/4286*ΩhrpB* meningococcal strains. **(A)** Brains of mice infected with 93/4286 or 93/4286Ω*hrpB* strain and sacrificed after 12, 24 and 48 h of infection were collected, homogenized, lysed, and subjected to immunoblotting. One mouse for each time point was infected with 93/4286, and two mice for each time point were infected with 93/4286Ω*hrpB*. Antibodies against vinculin, PARP-1 or TNF-α were used and anti-tubulin antibody was used as loading control. **(B–G)** Densitometric analysis quantification is reported as fold change of values of infected brains compared to values of non-infected brains. For mice infected with the 93/4286Ω*hrpB* the expression is reported as a scatter dot plot ± SD of values obtained with two mice infected at the same time point. Two-way ANOVA with Dunnett test was performed (*p<0.05, **p<0.01).

Then, we monitored the amount of pro-inflammatory cytokines interleukin-1β (IL-1β) (**Figures 4A, D**), IL-18 (**Figures 4A, E**) and tumor necrosis factor-α (TNF-α) (**Figures 5A, F**) during infection. Indeed, the increase in IL-1β and IL-18 which results from the processing of the immature forms of these interleukins by caspase-1 and are released through membrane pores formed by oligomerized N-terminal fragments of gasdermin-D, is an important end point of activation of the pyroptosis pathway (Jorgensen and Miao, 2015), while TNF−α is a pleiotropic cytokine that is produced in response to Toll-like receptor stimulation (Kalliolias and Ivashkiv, 2016; Parameswaran and Patial, 2010). Consistent with the trend of caspase-1 activation, we observed a strong decrease of IL-1β mature form in brains of mice infected with 93/4286*ΩhrpB* at each time point (**Figures 4A, D**) with a reduction of 72,7% ± 0.20 at 12 h,75,1% ± 0.03 at 24 h and 82,6% ± 0.04 at 48 h compared to 93/4286 infected tissue.

**Figure 6:**
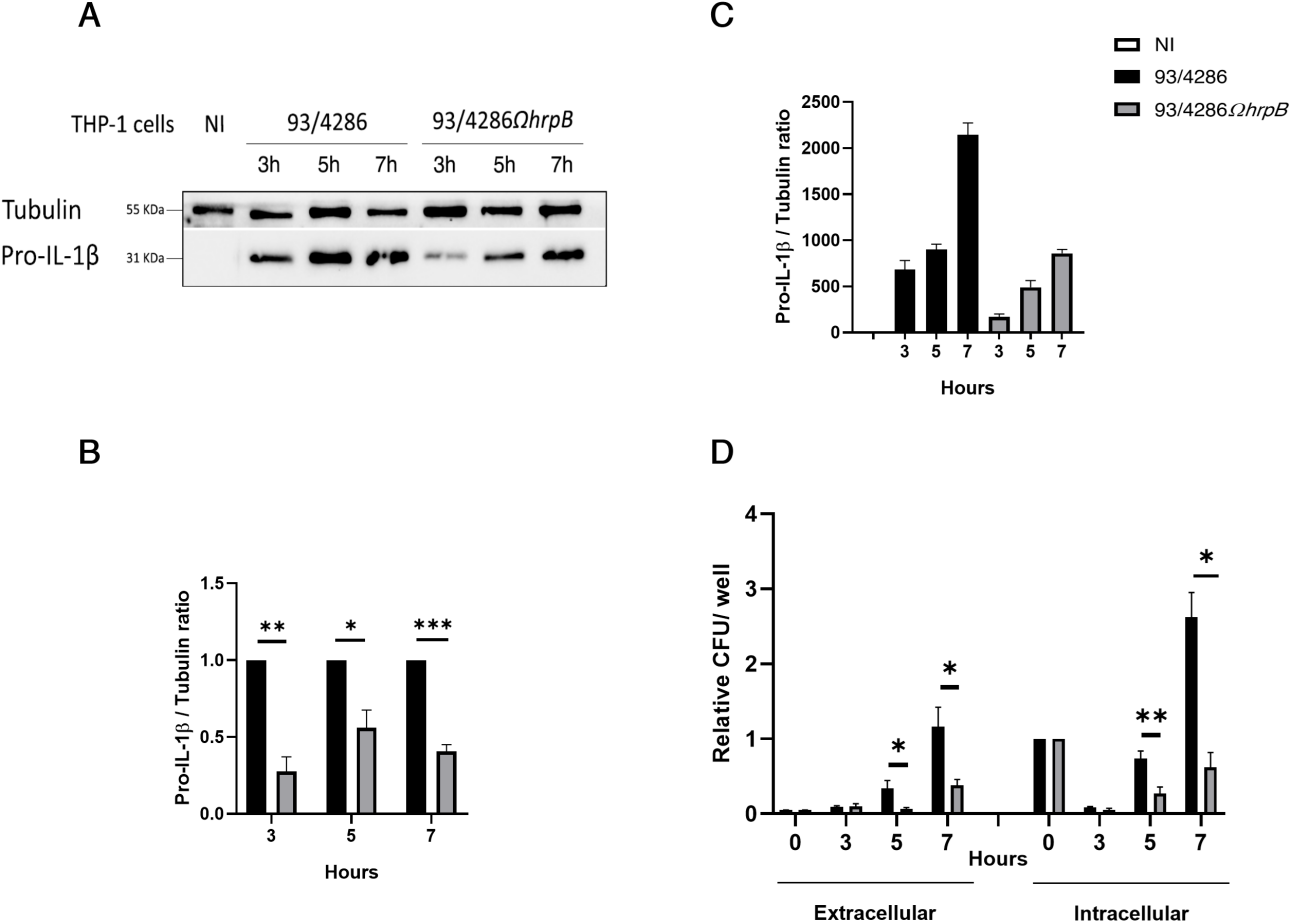
Intracellular replication and exit of *N. meningitidis* 93/4286 and 93/4286*ΩhrpB* strains from THP-1 macrophages and interleukin-1β (IL-1β) expression in cells after infection. **(A)** THP-1 cells were infected or not with 93/4286 or 93/4286*ΩhrpB* strain for 3, 5 and 7 h, lysed and subjected to immunoblotting. Antibodies against IL-1β and tubulin were used. **(B, C)** Densitometric analysis quantification reported as fold change of values of infected cells compared to values of non-infected cells or cells infected with 93/4286*ΩhrpB* strain compared to 93/4286 strain. Values are the mean ± SEM of three independent experiments, student t test has been performed. (*p<0.05, **p<0.01, ***p<0.001). **(D)** Result of gentamycin protection assay. Relative extracellular and intracellular CFU of 93/4286 and 93/4286*ΩhrpB* meningococci after 3, 5 and 7 h of infection in THP-1 cells. Values are the mean ± SDs of at least three independent experiments (*p<0.05, **p<0.01, ***p<0.001).

IL-1β expression was also evaluated in THP-1 macrophages after infection with the same strains for 3, 5 and 7 h; we were able to detect pro-IL1β only after infection with a reduction of 72% ± 0.09 after 3 h, 43% ± 0.1 after 5 h and 59% ± 0.04 after 7 h in cells infected with 93/4286*ΩhrpB* compared to 93/4286 strain (**Figures 6A–C**). At the same time points, the IL-1β expression was correlated with the growth/survive ability of the wild-type strain and *hrpB* mutant in THP-1 macrophages. Specifically, THP-1 macrophages were infected with meningococcal strains for 1 h and extracellular and intracellular bacterial viability was assessed, after gentamicin treatment (time 0 h). Results demonstrated that the *hrpB* mutant strain has significantly reduced ability to exit/replicate in the extracellular milieu, compared to the wild-type strain (**Figure 6A**). Intracellular replication/survival was markedly decreased in both strains during the first 3 h after infection, as normally occurs in meningococcal, and also in gonococcal invasion (Williams et al., 1998; Talà et al., 2008a, 2014; Colicchio et al., 2015). The number of recoverable CFU from cells started to increase from 5 up to 7 h post gentamicin treatment, showing significative differences between the two strains. Specifically, the number of viable intracellular bacteria of the wild-type strain increased about 2.5-fold at 7 h, compared with the initial value (time 0 h); in contrast, the number of recoverable CFU of the *hrpB* mutant did not reach the initial value (**Figure 6A**). It may be noted that similar results were previously observed in HeLa cells with strain B1940Ω*hrpB*, an *hrpB* mutant constructed from the serogroup B strain B1940 (Talà et al., 2008b), indicating that genetic inactivation of *hrpB* leads to a severe deficit in intracellular growth/survival regardless of meningococcal genetic background and host cell type.

IL-18 also showed a reduction in tissue infected with *hrpB* defective mutant strain compared to wild-type and in particular of 55% ± 0.05 at 12h, 53% ± 0.01 at 24 h and 55% ± 0.1 at 48 h (**Figures 4A, E**). TNF-α, on the contrary, could be detectable in both 93/4286- and 93/4286Ω*hrpB*-infected mice (**Figure 5A**) about at the same levels during the time course (**Figures 5A, F**). Regarding cleavage of PARP1, we detected cleavage fragments of approximately 70, 60, 30 and 18 kDa, abundant especially in infected brains at 12 h, and to a much lesser extent at 24 and 48 h (**Figures 5A–E**). At 12 h these fragments were slightly more abundant in mice infected with 93/4286 compared to those infected with 93/4286Ω*hrpB*, while at later time points (48 h) the opposite behavior was observed (**Figures 5A–E**).

In these experiments, we also used an antibody against vinculin, initially used as a reference protein in the western blot experiments. Unexpectedly, we found the vinculin (124 kDa) was cleaved during the infection into a 95 kDa fragment (**Figures 5A, G**). In fact, as far as we know, to date there is no evidence of this phenomenon during meningococcal infection. Comparatively, at the corresponding time points, we did not observe difference between 93/4286 and 93/4286Ω*hrpB* infected mice, while we observed a reduction of the vinculin cleavage in brains infected with 93/4286Ω*hrpB* after 24 and 48 h (by 80% ± 0.009 and 87% ± 0.001, respectively) compared to brains infected with 93/4286 while no changes after 12 h of infection (**Figures 5A, G**). Possible explanations and implications of vinculin cleavage are discussed below.

**Figure 7:**
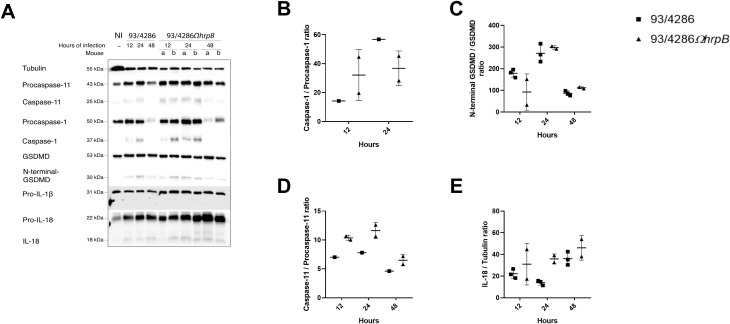
Pyroptosis markers expression in mouse spleen after infection with either 93/4286 or 93/4286Ω*hrpB* meningococcal strains. **(A)** Mice were infected with 93/4286 or 93/4286Ω*hrpB* strain and sacrificed after 12, 24 and 48 h of infection. Three mice for each time point were infected with 93/4286, and two mice for each time point were infected with 93/4286Ω*hrpB*. Spleens were collected, homogenized, lysed, and analyzed by immunoblotting using antibodies against caspase-11, caspase-1, gasdermin-D (GSDMD), interleukin 1β (IL-1β) and IL-18. Anti-tubulin antibody was used as a loading control. **(B–E)** Densitometric analysis quantification is reported as fold change of values of infected spleen compared to values of non-infected tissue. Values are reported as scatter dot plot ± SD. Two-way ANOVA with Dunnett test was performed.

In contrast to what was observed in the brain, however, no significant differences in the activation of pyroptotic pathways were found in the spleen of mice infected with the 93/4286 or 93/4286Ω*hrpB* strain (**Figure 7**; **Supplementary Figure S3**). In the spleen, the mice infected with the wild type and mutant strains showed a similar pattern, with an increase in the amount of caspase-1 and caspase-11 at 24 h, and a decrease in the amounts of caspase-1 and caspase-11 and respective procaspase-1 and procaspase-11 at 48 h (**Figures 7A, B, D**). Compared to 93/4286-infected mice, a small increase in the amount of caspase-11 (by 49,15% ± 0,12) and, conversely, a small decrease in the amount of caspase-1 (by 35,31% ± 0,15) was observed at 24 h in spleens of 93/4286Ω*hrpB*-infected mice. GSDMD was activated in the spleen with a peak of activation at 24 h with no differences between the two strains (**Figures 7A, C**). Even if active forms of caspase-1, caspase-11 and GSDMD were detected, only a small fraction of pro-IL-18 was cleaved into mature form in the spleen, and no mature IL-1β was detected in this tissue through western blot analysis (**Figures 7A, E**).

## 4 Discussion

In this study, the availability of the mouse model of meningococcal meningitis gave us the opportunity to compare and contrast the results obtained with *in vitro* cell infection model with those obtained in an *in vivo* model, regarding the role of the HrpA/HrpB TPS system as a virulence determinant and the ability of meningococcus to induce pyroptotic pathways and the underlying mechanisms. The results of this study, although obtained with a limited number of animals, demonstrated that the HrpA/HrpB TPS system is required for virulence in the mouse model as we observed 100% survival of animals challenged with 10^7^ CFU of the mutant 93/4286Ω*hrpB* strain and 50% survival in mice inoculated with 10^6^ CFU of the wild-type 93/4286 strain (**Figure 2A**). The difference in virulence between the wild-type and the mutant strain resulted in a reduced replicative capacity in the brain and a reduced survival in the spleen and liver (**Figure 3**), although the mutant strain was still able to disseminate into the spleen and liver. Thus, the *hrpB* mutant retained the ability to spread systemically, albeit to a lower level than the wild-type strain, but was unable to kill the animals. Furthermore, no significant differences in the activation of pyroptotic pathways were found in the spleen of mice infected with the 93/4286 or 93/4286Ω*hrpB* strain (**Figure 7**; **Supplementary Figure S3**). Since in our experimental model the death of the animal occurs as a result of brain infection and inflammation while the bacteremia is only secondary to the diffusion of the bacteria from the *choroid plexus* to the bloodstream using a reverse path (Colicchio et al., 2019), it can be deduced that the animals infected with the *hrpB* mutant did not die because the damage to the brain was not capable of causing death. Therefore, *hrpB* inactivation produced effects primarily in the mouse brain in our experimental meningitis model.

We do not know why the mutant strain has poor replicative capacity/survival in the mouse, but we speculate that the same mechanisms observed in *in vitro* cell infection models may also be implicated in the mouse model. In particular, in different cell types, HrpA plays a role in the escape from the internalization vacuole into the cytoplasm thereby promoting bacterial intracellular survival/growth. In contrast to wild-type bacteria, HrpA/HrpB-defective mutants are confined to late endocytic vacuoles and killed by lysosomes (Talà et al., 2008b). Although we have no direct evidence, we hypothesize that the reduced ability of the *hrpB* mutant to replicate/survive in mouse tissues compared to the wild-type strain may be caused by the reduced ability to replicate/survive in the cells (**Figure 6D**) (Talà et al., 2008b). In fact, even if in the mouse model used, the meningococci are inoculated intracisternally, the animals develop both meningitis and disseminated infection, and this presupposes that the bacteria must cross the cellular barriers to spread in the blood and reach the peripheral organs, including the spleen and the liver (Colicchio et al., 2009, 2019). Furthermore, in the mouse brain the meningococci seem to have an intracellular localization, particularly in the cells of the *corpus callosum*, in the ependymal cells lining the ventricles and in epithelial cells of the *choroid plexus*, that can be used to spread systematically using a reverse path (Colicchio et al., 2009, 2019).

Cell death processes often determine the outcome of microbial infection. In fact, although these processes are part of the host’s defense mechanisms, determining in many cases the removal of the pathogen, the type of cell death (apoptosis, pyroptosis, necroptosis) can be beneficial or harmful for the host influencing the extent of inflammation and the progression of the infection (Labbé and Saleh, 2008). Like pyroptosis, necroptosis, initially defined as programmed necrosis (Degterev et al., 2005), is a pro-inflammatory cell death process that mimics the characteristics of apoptosis and necrosis, and many bacterial pathogens have recently been shown to induce and modulate this process in phagocytic cells to establish persistent inflammation (Bonhomme et al., 2023; Hao et al., 2021; Kundu et al., 2022; Vu et al., 2024; Yu et al., 2024). Apoptosis, pyroptosis and necroptosis (programmed necrosis) are characterized by the activation of caspases and the production of cytokines through partially shared pathways and are therefore interconnected leading to the establishment of the concept of PANoptosis (Sun et al., 2024; Wang and Kanneganti, 2021; Sundaram et al., 2024). The balance between these processes is very critical for the outcome of a microbial infection, and depends on both microbial and host factors. Some pathogenic bacteria have the ability to differently induce apoptosis, pyroptosis and necroptosis depending on the host microenvironment and cell type (Wang and Kanneganti, 2021). On these aspects, however, there is only little information on meningococcus, which may be responsible for an invasive disease characterized by intense inflammation.

The results with *in vitro* cell infection models also demonstrate that, by affecting the meningococcal trafficking within different cell types, the HrpA/HrpB TPS system modulates the balance between apoptosis and pyroptosis in favor of the latter (Talà et al., 2022). Regarding apoptosis, the meningococcus may suppress apoptosis by translocating the porin PorB into the mitochondrial membrane by an unknown mechanism. After translocation, PorB was shown to prevent mitochondrial membrane depolarization by interacting with voltage-dependent anion-selective channel (VDAC) and probably by interfering with the contact sites between the outer and inner mitochondrial membranes (Massari et al., 2000, 2003, 2010). The information obtained with HrpA/HrpB TPS-defective mutants in *in vitro* cell infection model (Talà et al., 2022) and the mouse model of meningococcal meningitis in the current study allows us to propose some missing pieces to this model.

After entry into the host cell, *N. meningitidis* exits the internalization vacuoles (Talà et al., 2008b, 2022). This process is favored by HrpA, with its carboxy-terminal lysin domain (Talà et al., 2022), although it also occurs to a lesser extent in *hrpA*-deficient bacteria (Talà et al., 2008b). In the cytoplasm, meningococci contact the tubulin cytoskeleton, an interaction probably favored by the architecture of capsular polysaccharides (Talà et al., 2014; Manno et al., 2021). They then dock at the dynein motor, thanks to the interaction between HrpA and DYNLT1 (Talà et al., 2022). Integrating the old model (Massari et al., 2000, 2003, 2010), it has been proposed that the HrpA-DYNLT1 interaction could promote PorB translocation into the mitochondrial membrane through different mechanisms, for example, either promoting the contact between intracellular bacteria and mitochondria on the tubulin cytoskeleton, or facilitating the interaction between mitochondria and meningococcal outer membrane vesicles (Talà et al., 2022), which carry high amounts of PorB and also contain TPS proteins (Lappann et al., 2013). The translocation of PorB to the mitochondria and its interaction with the VDAC would determine the inhibition of the intrinsic pathway of apoptosis (Massari et al., 2000, 2003, 2010). This model on apoptosis inhibition appears to be consistent with our results with the animal model, as we observed that the caspase-3 (**Figure 4**), an “executioner” caspase on which both intrinsic and extrinsic apoptotic pathways converge (Vaughan et al., 2002), increased its expression in the mouse brain after the meningococcal infection but was not activated.

Regarding pyroptosis, results with the *in vitro* cell infection models demonstrated that the meningococcus is able to induce pyroptosis, a form of pro-inflammatory cell death that relies on activation of several caspases (Talà et al., 2022). The induction of pyroptosis was observed in all cell lines analyzed including human epithelial HeLa cells, mouse NSC34, a hybrid cell line derived from the fusion of mouse motor neuron-enriched embryonic spinal cord cells with mouse neuroblastoma cells (Cashman et al., 1992), and Human Brain Microvascular Endothelial Cells (HBMEC) cell line (Eigenmann et al., 2013), an established model to study the interaction of the meningococcus with the blood-brain barrier (Coureuil et al., 2009; Join-Lambert et al., 2010).

There are two known pathways leading to pyroptosis: i) the canonical inflammasome: the classical caspase-1-dependent pathway; ii) the non-canonical inflammasome: the non-classical pathway that depends on the activation of caspase-4/5 (caspase 11 in mice). The canonical inflammasome pathway is activated by a wide range of infectious and non-infectious stimuli, and relies on platforms called “inflammasomes”, which are multi-protein signaling complexes that assemble in response to noxious stimuli such as damage-associated molecular patterns (DAMPs) and pathogen-associated molecular patterns (PAMPs) (Huang et al., 2019). In contrast to the canonical pathway, the non-canonical inflammasome pathway involves direct recognition of cytosolic LPS/LOS by the CARD of caspase-4 and caspase-5 in humans and caspase-11 in mice (Yang J et al., 2015). These pathways converge in the activation of gasdermin-D through the cleavage of the 53 kDa gasdermin-D to form a N-terminal fragments of gasdermin-D of 31 kDa that oligomerizes to form membrane pores (Huang et al., 2019; Man et al., 2017; Shi et al., 2017). In addition to cleave gasdermin-D, caspase-1 is known to process two pro-inflammatory cytokines, pro-interleukine-1β (pro-IL-1β) and pro-interleukine-IL-18 (pro-IL-18) into biologically active IL-1β and IL-18 (Jorgensen and Miao, 2015).

It has been proposed that after exiting the internalization vacuole meningococci induce the pyroptosis by exposing and/or releasing the LOS into the cytosol through the non-canonical inflammasome pathway (Talà et al., 2022). In fact, a strong activation of caspase-11 was observed in NSC-34 mouse neuronal cells infected with wild-type meningococci and a much lower activation in cells infected with bacteria defective in HrpA/HrpB TPS which facilitates the exit of bacteria from the internalization vacuole (Talà et al., 2022). The reduction of caspase 11 activation by TPS-defective HrpA/HrpB meningococci may be determined by their reduced ability to escape from the internalization vacuole, that would allow them to expose or release LOS into the cytosol, and/or by their reduced ability to survive into cells by avoiding lysosomal killing (Talà et al., 2022). Consistent with this finding, in this study we observed a strong activation of caspase-11 in the mouse BALB/c brain infected with the wild-type 93/4286 and a weaker activation in mice infected with the 93/4286Ω*hrpB* strain (**Figure 4**; **Supplementary Figure S2**). It can also be seen that the decrease in caspase-11 activation by the *hrpB* mutant correlates over time (**Figures 4A, B**) with a decrease in CFU in the brains of infected mice (**Figure 2A**). This finding may suggest that the reduced survival capacity in the brain was primarily responsible for decreased inflammasome activation rather than the reduced ability to escape the internalizing vacuole, although further experiments are needed to clarify this issue in the mouse model. On the other hand, in the cellular infection model the reduced survival capacity is closely related to the reduced ability to escape the internalization vacuole (Talà et al., 2008b).

In the brains of BALB/c mice we could also observe an activation of caspase-1 after infection with the wild-type 93/4286, while a much lower activation was observed in the brains of mice infected with the 93/4286Ω*hrpB* mutant (**Figures 4A, C**). This result is in agreement with previous findings showing a strong activation of caspase-1 in NSC-34 mouse neuronal cells infected with wild-type meningococci and a much lower activation in cells infected with *hrpA*-defective bacteria (Talà et al., 2022), and may suggest an involvement of the canonical inflammasome pathway. During meningococcal infection, caspase-1 activation could occur via the canonical pathway, following bacterial infection and release of pathogen-associated molecular patterns into the cytosol, and assembly of “inflammasome” platforms. However, it can be also noted that caspase-1 can be recruited to the non-canonical pathway by an unknow mechanism relying on caspase-11 activation by LPS/LOS, gasdermin-D processing and nucleotide-binding oligomerization domain–like receptor pyrin domain–containing-3 (NLRP3) activation to promote caspase-1 activation and subsequent cleavage and release of IL-1β and IL-18 (Kayagaki et al., 2011, 2015). In fact, it has been also shown that upon intracellular LPS stimulation, caspase-11 also cleaves Pannexin-1, mediating ATP release and K^+^ efflux to trigger activation of the NLRP3 inflammasome (Yang D et al., 2015).

As a consequence of caspase-1 and/or caspase-11 activation, gasdermin-D was activated in the brains of BALB/c mice infected with the wild-type 93/4286 and, to a lesser extent, in the brains of mice infected with the 93/4286Ω*hrpB* mutant in which caspase-1 and caspase-11 were less activated (**Figures 4A, F**). Consistent with the trend of caspase-1 activation, we were able to detect a greater amount of IL-1β and IL-18 in brains of mice infected with 93/4286 compared to those infected with 93/4286Ω*hrpB* (**Figures 4A, D, E**). Of note, gasdermin-D activation could not be observed in cells infected *in vitro* with meningococci (Talà et al., 2022) and this finding was attributed to the activation of caspase-3, an “executioner” caspase of apoptosis, which has been shown to suppress gasdermin-D cell lysis during extrinsic apoptosis (Chen et al., 2019). Conversely, caspase-3 was not activated in the BALB/c brain, so we could detect gasdermin-D activation after meningococcal infection (**Figures 4A, F**). Since gasdermin-D activation has a crucial role in the effector phase of pyroptosis, it would be interesting to analyze the virulence of wild-type strain 93/4286 and strain 93/4286Ω*hrpB* in gasdermin-D-defective mouse models (Xu et al., 2022).

Caspase-7, another “executioner” caspase of apoptosis, which at variance with caspase-3 is processed by caspase-1 (Lamkanfi et al., 2008), was activated in the mouse brain infected with the wild-type strain and, to a much lesser extent, with the *hrpB* mutant (**Figures 4A, G**). Notably, caspase-7 has reported roles not only in apoptosis (Lamkanfi and Kanneganti, 2010; Brentnall et al., 2013), but also in pyroptosis as it is involved in inflammasome signaling (Lamkanfi and Kanneganti, 2010; Erener et al., 2012). Upon caspase-1 activation, caspase-7 moves to the nucleus to enhance the expression of a subset of genes under the control of NF-κB (Erener et al., 2012). Moreover, gasdermin-D and caspase-7 have been reported as key mediators downstream inflammasome activation during *Legionella pneumophila* infection (Goncalves et al., 2019). This information is relevant because in the brain of infected mice caspase-7 and gasdermin-D showed a similar activation pattern by western blot (**Figures 4A, F, G**).

In our study we also found that the vinculin was cleaved during the infection into a 95 kDa fragment (**Figures 5A, G**). At later time points (24 and 48 h) this cleavage was more pronounced in the brains of animals infected with 93/4286 wild-type strain than in those infected with the 93/4286Ω*hrpB* mutant, consistent with a greater severity of disease caused by the wild-type strain. To date there is no evidence of this phenomenon during meningococcal infection. However, there is evidence that vinculin is proteolyzed into the 95 kDa fragment by calpain during platelet aggregation (Serrano and Devine, 2004). After proteolysis, the 95 kDa vinculin fragment shifts cellular compartments from the membrane skeletal fraction to the cortical cytoskeletal fraction contributing to the cytoskeletal remodeling of aggregating platelets (Serrano and Devine, 2004). This remodeling and the underlying mechanisms may play a crucial role during intravascular disseminated coagulation of invasive meningococcal disease and will also merit future investigation. The reduction in vinculin cleavage in the 93/4286*ΩhrpB* mutant may reflect a reduced ability of the mutant to interact with platelets and activate platelet aggregation compared to the wild-type 93/4286 strain. This interaction could occur through the fibrinogen receptor (Deppermann and Kubes, 2016; Hamzeh-Cognasse et al., 2015) since, among the interactors of HrpA, the fibrinogen alpha chain (FGA) has emerged (Talà et al., 2022). Furthermore, it may be also hypothesized that the interaction of the bacteria with the fibrinogen may also play a role in mouse brain invasion as shown in experimental meningitis models with group B Streptococcus (Beninati et al., 2019).

**Figure 8:**
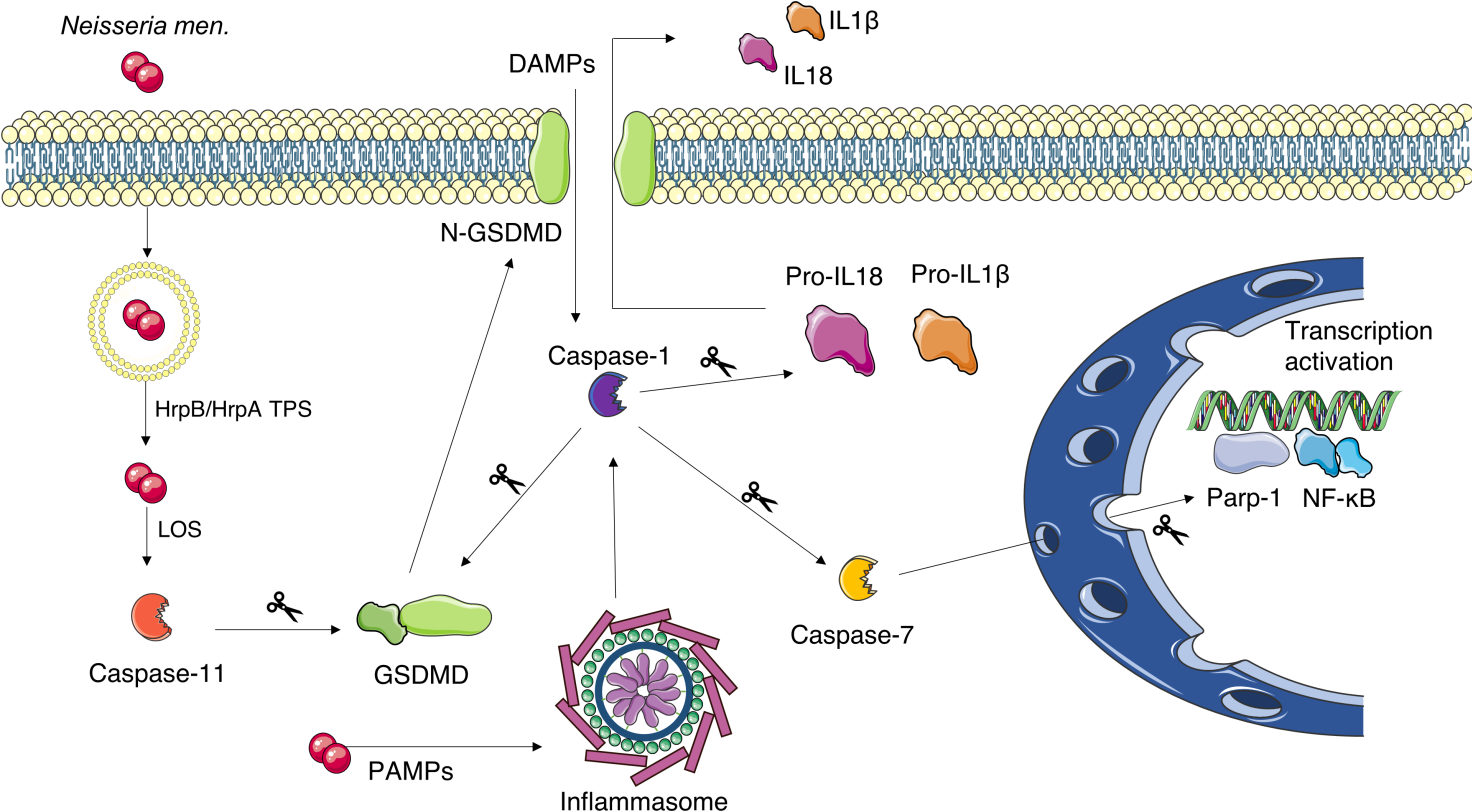
Schematic representation of the proposed pyroptosis activation mechanism in *N. meningitidis*-infected mouse brains. The bacterium gains the cytosol of the cell by exit the internalization vacuole. Vacuole escape is favored by HrpB/HrpA TPS. The subsequent exposure of the LOS activates caspase-11, while PAMPs activate the inflammasome and, in turn, caspase-1. Caspase-1 can be also activated by other/additional mechanisms including cleavage of Pannexin-1 by caspase-11, which results in ATP release, K^+^ efflux, and NLRP3 inflammasome activation (not shown). Both caspase-1 and caspase-11 can cleave gasdermin-D (GSDMD) releasing its N-terminal peptide. The latter forms pore in the cell membrane through which caspase-1-activated IL-1β and IL-18 are secreted. Caspase-1 can also cleave caspase-7 which is able to enhance the expression of pro-inflammatory genes under the control of NF-κB. This Figure was created by the use of Servier Medical Art, provided by Servier, licensed under a Creative Commons Attribution 3.0 unported license.

Activation of the above-described pyroptotic pathways in the mouse model could be involved in the characteristic inflammation of invasive meningococcal disease also in the human host, and the HrpA/HrpB TPS may play a role in the activation of this pathway. A schematic representation of the activation of these pyroptotic pathways is depicted in **Figure 8**. By affecting the meningococcal trafficking within infected cells, the HrpA/HrpB TPS system appears to modulate the induction of these pyroptotic pathways and to play a pivotal role in the induction of invasive meningococcal disease in the murine model. However, it should be noted that important differences in the induction of the pyroptotic pathways were found in the brain and spleen along with a different effect of *hrpB* inactivation in the two tissues. Such behavior is consistent with the observation that the ability of pathogenic bacteria to differently induce apoptosis, pyroptosis and necroptosis depends on the host tissue and cell type (Wang and Kanneganti, 2021).

These results also open the possibility to evaluate the use of pyroptosis inhibitors in adjunctive therapy of meningococcal meningitis and invasive meningococcal disease. Among them, specific caspase-1 inhibitors could be evaluated, including VX-765 (belnacasan) for which phase II clinical trials for the treatment of psoriasis have been completed, and VX-740 (pralnacasan) for which phase II clinical trials are in progress for the treatment of rheumatoid arthritis and osteoarthritis (Dhani et al., 2021). Broad-spectrum caspase inhibitors could also be evaluated, such as VX-166, which is active against caspase-1, caspase-3 and caspase-7, and has been proposed for the treatment of sepsis and endotoxic shock (Dhani et al., 2021; Di Salvo et al., 2019). Finally, IZD174 (Inzomelid), an NLRP3 inhibitor, should be mentioned for its ability to cross the blood-brain barrier (Coll et al., 2022). Phase II clinical trials for the treatment of cryopyrin-associated periodic syndrome have been completed for this inhibitor (Coll et al., 2022). It is also important to underline that many pyroptosis inhibitors are subject to clinical trials for various pathologies, but none of these are subject to clinical trials for the treatment of sepsis or invasive bacterial diseases, such as meningococcal disease, due to the lack of sufficient studies on animals. We hope that the current study aims to help fill this information gap to find more effective therapeutic solutions to a devastating disease.

## Data Availability

The raw data supporting the conclusions of this article will be made available by the authors, without undue reservation.
