# Supplementary material for: Neisseria meningitidis activates pyroptotic pathways in a mouse model of meningitis: role of a two-partner secretion system
Source: Front Cell Infect Microbiol. 2024 Sep 23;14:1384072. doi: 10.3389/fcimb.2024.1384072 (PMC11456522; doi:10.3389/fcimb.2024.1384072)
Supplement: Supplementary file 1 [file DataSheet1.docx]

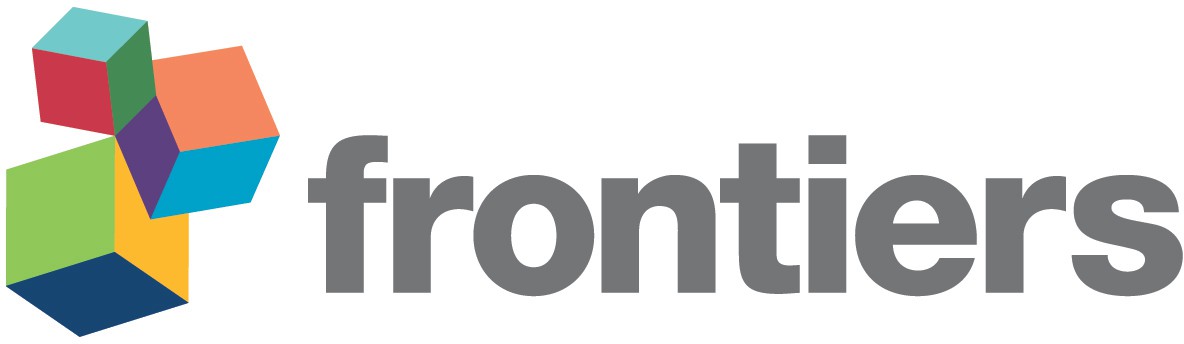


Supplementary Material

***Neisseria meningitidis* activates pyroptotic pathways in a mouse model of meningitis: role of a two-partner secretion system**

# Chiara Pagliuca^1^, Roberta Colicchio^1,^ *, Silvia Caterina Resta^2^, Adelfia Talà^2^, Elena Scaglione^1^, Giuseppe Mantova^1^, Leonardo Continisio^1,3^, Caterina Pagliarulo^4^, Cecilia Bucci^5^, Pietro Alifano^5^ ^#^ and Paola Salvatore^1, 6,7^ ^#,^ *

^1^Department of Molecular Medicine and Medical Biotechnologies, University of Naples “Federico II”, Naples, Italy

^2^ Department of Biological and Environmental Sciences and Technologies, University of Salento, Lecce, Italy

^3^ PhD National Programme in One Health approaches to infectious disease and life science research, Department of Public Health, Experimental and Forensic Medicine, University of Pavia, Pavia, Italy

^4^Department of Science and Technology, University of Sannio, Benevento, Italy

^5^ Department of Experimental Medicine, University of Salento, Lecce, Italy

^6^ CEINGE-Biotecnologie Avanzate Franco Salvatore s.c.ar.l., Naples, Italy

^7^ Task Force on Microbiome Studies, University of Naples “Federico II”, Naples, Italy

**#** These authors contributed equally

*Corresponding author: e-mail address:

roberta.colicchio@unina.it; [psalvato@unina.it](mailto:psalvato@unina.it)

Supplementary Material

# 1 Supplementary Figures


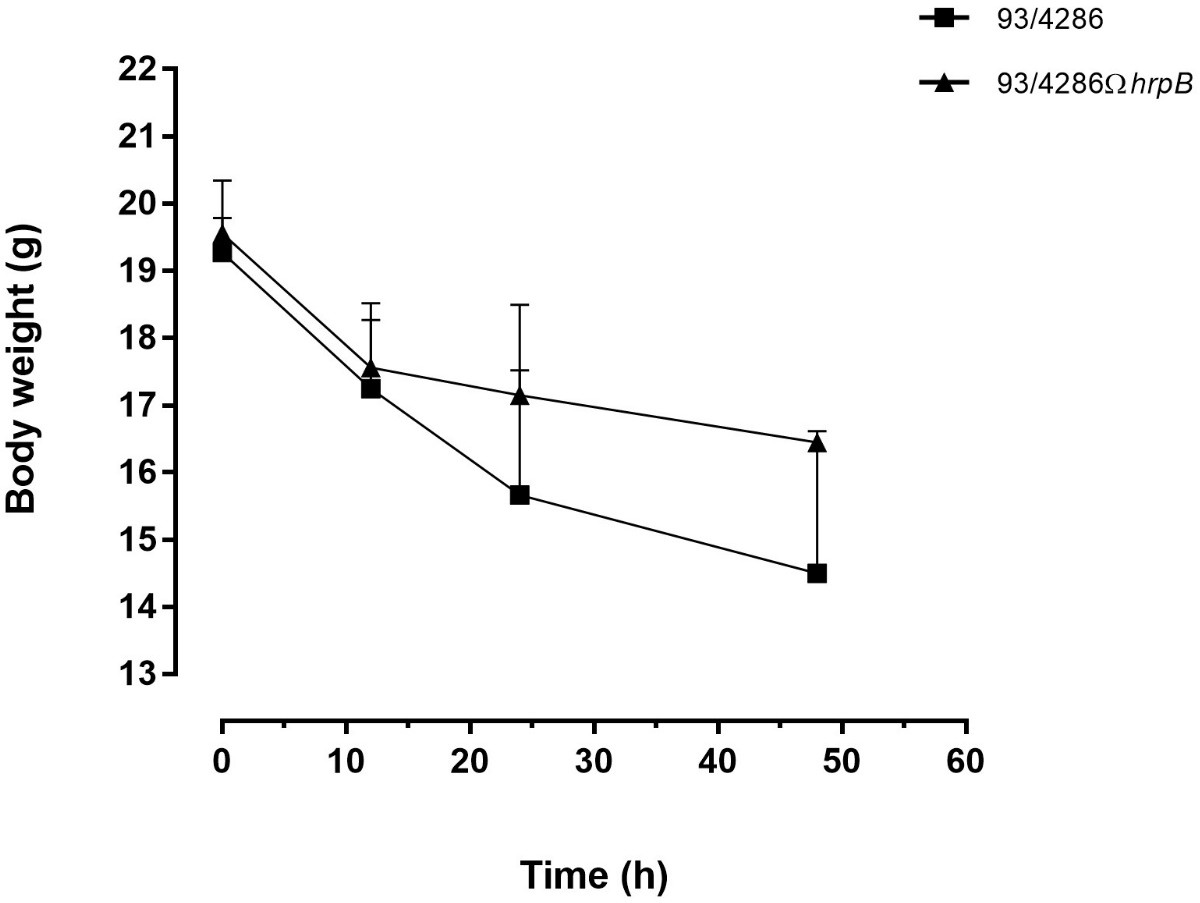


**Supplementary Figure S1** Variation of body weight after meningococcal infection of BALB/c mice with 10^6^ CFU/mouse of 93/4286 strain (squares) or 10^7^ CFU/mouse of the isogenic 93/4286Ω*hrpB* mutant (triangles). Results are indicated as mean ± SD.


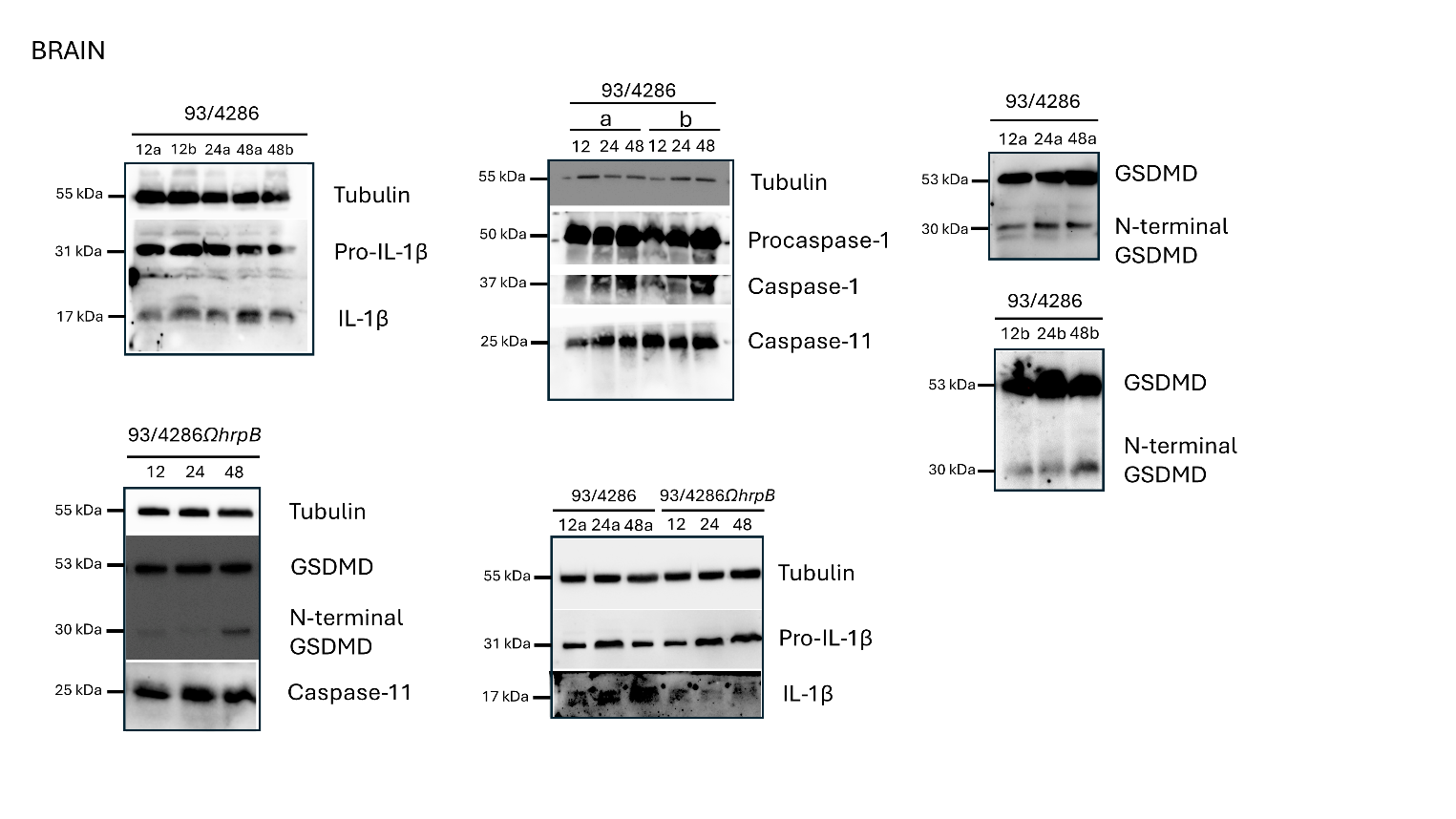


**Supplementary Figure S2** Western blots of pyroptotic markers in brain tissue from mice infected with 93/4286 or 93/4286*ΩhrpB* strains for 12 h, 24 h or 48 h. Two mice for each time point (n = 6 mice) were infected with 93/4286 (a and b) and one mouse with 93/4286*ΩhrpB* for each time point (n =3 mice)*.* Antibodies against IL-1β, Caspase-1, Caspase-11 and Gasdermin D (GSDMD) were used. Anti-tubulin antibody was used as loading control. Densitometric analysis quantification and Two-way ANOVA test were performed and showed in Figure 4.


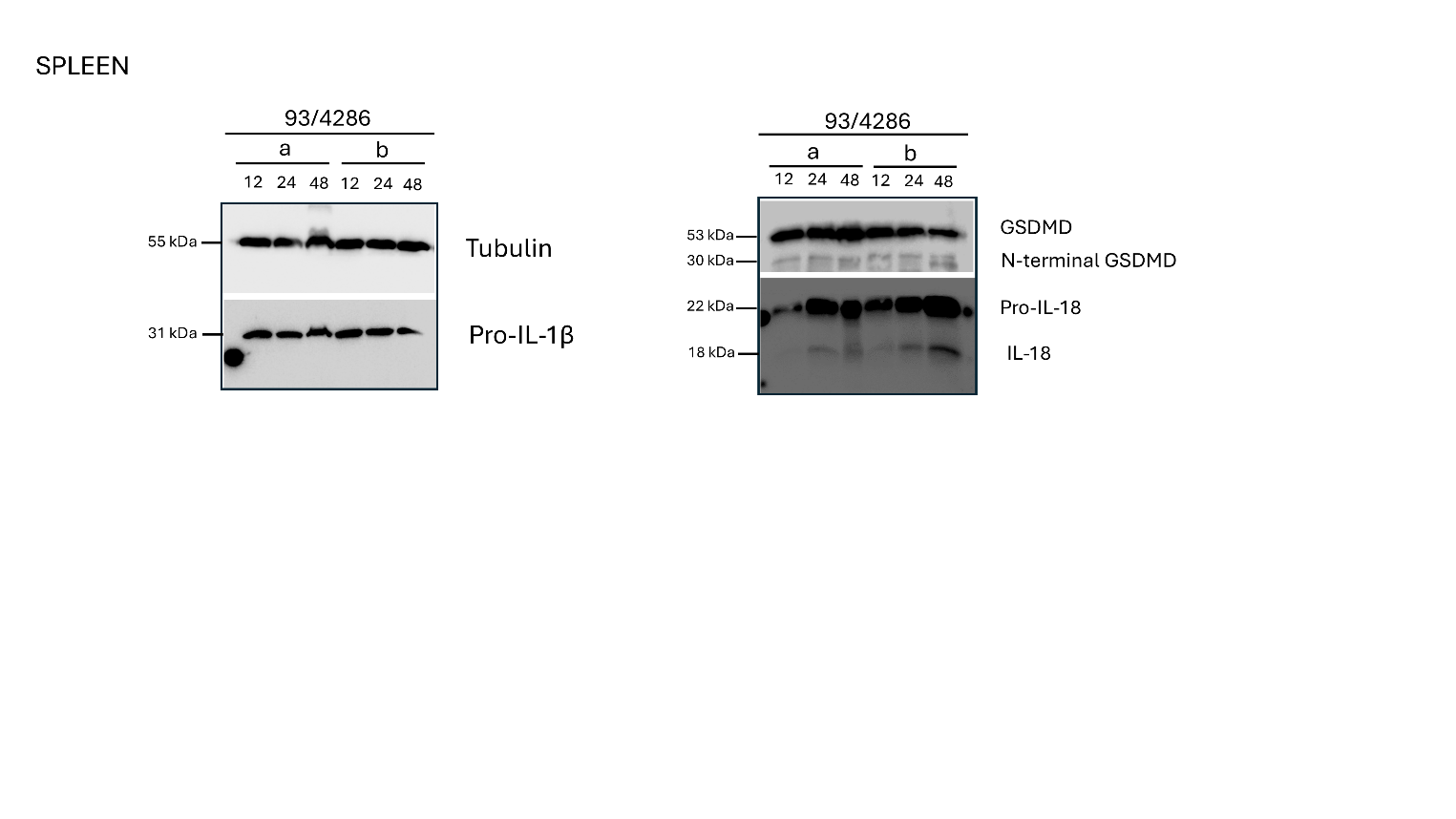


**Supplementary Figure S3** Western blots of pyroptotic markers in spleen tissue from mice infected with 93/4286 strain for 12 h, 24 h or 48 h. Two mice for each time point were infected with 93/4296 strain (a and b) (n =6 mice). Antibodies against IL-18 and Gasdermin D (GSDMD) were used. Anti-tubulin antibody was used as loading control. Densitometric analysis quantification and Two-way ANOVA test were performed and showed in Figure 7.
